# Variation in Interactive Gestures by Visual Occlusion and Topic Complexity: Evidence for a Subconscious Theory of Gesture

**DOI:** 10.1111/cogs.70195

**Published:** 2026-03-10

**Authors:** T. R. Williamson, Kristofer Kinsey, Anna E. Piasecki

**Affiliations:** ^1^ Brain, Language, and Behaviour Laboratory University of the West of England; ^2^ Southmead Hospital, North Bristol NHS Trust

**Keywords:** Interactive, Gesture, Conversation complexity, Theory of mind, Subconscious

## Abstract

Gestures are often categorized into types: iconics, metaphorics, and pantomimes (having representational relationships with spoken semantics), deictics (i.e., pointing), emblems (having their own conventional meaning), and beats (temporally coinciding with spoken content for emphasis). These originate from research often involving unnaturalistic paradigms where participants’ gestures during responses (e.g., retelling a narrative) are recorded. Approaching these types implicitly requires a stance on why we gesture; a conscious aim to communicate or an unconscious effort to orchestrate speech. Focus on them has led to the understudying of the interactive role gestures can play, where intersubjective acknowledgment and information transfer are central. This paper has two main aims: to profile the interactive role of gesturing as a proportion of all gesturing and to investigate its relevance for why humans gesture. We report data from 48 28‐min dyadic conversations with a naïve participant and a confederate, varying interlocutors’ gesture visibility and conversation complexity. Our first, preregistered, analysis coded for the six traditional gesture categories, which resulted in ∼28% being uncodeable. Our second analysis asked whether these were interactive, which accounted for nearly 90% of uncoded gestures and a quarter of the entire database. Occluding gesture visibility significantly decreased the amount of interactive gestures participants made, resulting from a drop in interactive gestures made during simple conversations; complex topic interactive gesture frequency is stable between visually available and occluded conditions. Our data support both philosophies, but advocate for a *sub*conscious account: that we gesture for the intrinsic motivations to express ourselves and to be understood.

## 1 Introduction

The scientific study of gesture is necessarily categorical. That is, the analysis of bodily movements that humans make when they express themselves must be categorized by an array of variables. In lay terms, we might suggest these include effector (which part of the body), form (what appearance the effectors take), motion (in what way the effectors’ position in space changes), and intention (what effect the form and/or motion of the effectors is intended to do have, be that intention conscious or not).

Kendon (2004a) sets out a comprehensive history of the categorization of gestures in academic study dating back to classical periods, yet the prevailing system descends from McNeill's (1992) quadripartite distinction. He advances the existence of representational gestures, of which two forms predominate in scientific analysis, plus beats and deictics. Representational gestures are those for which effectors’, such as hands’, forms in some way depict that to which the speaker refers. There are two kinds: where hands attempt to literally embody the speaker's referent (iconic gestures), and where the hands capture the source domain (*qua* Lakoff & Johnson, 1980) of a metaphor whose target is externalized linguistically (metaphoric gestures^1^). Beats are those gestures whose motions temporally coordinate with suprasegmental or semantic landmarks in speech (e.g., the politician's baton hand landing on every word). Deictics are simply pointing. Additionally, studies occasionally include two other gesture types. One is the emblem (Ekman & Friesen, 1969), a gesture whose form or motion is conventionally associated with specific meanings (e.g., thumbs up for “good,” palm facing away, and waving hand for “hello”). Another is the pantomime (Wundt, 1973), which we can consider another kind of representational gesture, where hands mimic the action of using a specific object or tool (e.g., pretending to grip a pole and moving side to side, mimicking sweeping). In almost all studies of gesture, these six types, to which we refer as “roles,” given that one handshape may play the *role* of many types depending on context, are the explananda.

Comparatively, little attention has been paid to the interactive role that gestures play. These gestures seem to have little communicative role, often having no inherent content or corresponding co‐speech semantics, instead pertaining to interlocutors’ thoughts and ideas. The earliest substantive work comes from Bavelas, Chovil, Lawrie, and Wade (1992), noticing that some gestures are characterized by motions of hands toward an interlocutor with forms of palms facing upward and fingers almost pointing at them. They identified four main roles of interactive gestures: citing the other's previous contribution; seeking agreement, understanding, or help; the delivery of new versus shared information; and events around the speaking turn. Such observations feed well into Kendon's (2004b, 2017) philosophy of gestures; that they are *pragmatic*. For Kendon, a pragmatic gesture is that which one intends to make with a communicative contribution (as in linguistic pragmatics, where pragmatic meaning is one a speaker intends; Grice, 1975). Importantly, a gesture having an interactive role is not equivalent to its being “pragmatic,” for the former refers to intentional effort to manage the discourse (e.g., its contents, its interlocutors, etc.; as in Bavelas, Chovil, Coates, & Roe, 1995; Wehling, 2017). In this way, any gesture can be pragmatic, but not every interactive gesture intentionally communicates as a default (*qua* Jaszczolt, 2005). This pragmatic perspective has proved popular from which to epistemologically begin studies of gesture (Bressem & Müller, 2014; Lopez‐Ozieblo, 2020; Payrató & Teßendorf, 2014), and could feasibly apply to all gesture roles mentioned in the previous paragraph, but, critically, is mutually exclusive. This is not to say that no work on interactive gestures exists: psycholinguistic (Curioni, Knoblich, Sebanz, & Sacheli, 2020; Holler, 2010; Kısa, Goldin‐Meadow, & Casasanto, 2024) and aphasiological (de Beer, de Ruiter, Hielscher‐Fastabend, & Hogrefe, 2019) investigations have probed their qualities and uses.

This idea that gestures are conscious intentional communicative acts contraposes McNeill's (1992, 2015), that they represent an orchestration of speech of which speakers are unconscious or unaware. One can only speculate whether the popularity of McNeill's (1992) classification system, which arose in the context of observational studies conducted in a narrative recall paradigm, led to the predominance of research into the types he identified, thus accidentally shifting focus away from the possibility of types not congruent with his philosophy.

In this study, we recorded British monolingual English participants in dyadic exchanges with a confederate across three stages, varying torso (thus gesture) visibility and topic complexity of conversation question prompts. Given that gestures exist primarily in the visual medium, many researchers have used gesture visibility as an independent variable. Early studies probed people's gestures as they communicated directions via intercom (Cohen, 1977; Cohen & Harrison, 1973); a paradigm complemented with more recent technologies like speaking on the telephone (Bavelas, Gerwing, Sutton, & Prevost, 2008). Gesture visibility during in‐person interaction has been achieved by including a partition within experimental designs (Alibali, Heath, & Myers, 2001; Emmorey & Casey, 2001). Studies leveraging computer‐aided interaction have successfully modulated partial visual occlusion, too (ter Bekke, Levinson, van Otterdijk, Kühn, & Holler, 2024; Trujillo, Levinson, & Holler, 2021). However, no study has both manipulated gestures’ visibility while carrying out *in‐person* interaction during a *naturalistic conversation* design, which limits these papers’ applicability to interpreting the findings herein.

Use of a confederate, and thus deception, requires some justification. Deception is often used in gesture experiments, perhaps due to the heightened confounding proprioceptive self‐awareness (of the hands) possible from informing participants about the study's relation to gesture (e.g., Zhang, Givvin, Sipple, Son, & Stigler, 2021) or a possible increase in gesture production relative to a baseline (Cravotta, Busà, & Prieto, 2019). This deception has also extended to concealing the role of a confederate in the experimental context to avoid biasing participants (Trujillo, Simanova, Bekkering, & Özyürek, 2018). Evidence shows that untrained confederates’ nonverbal behavior can bias participants’ responses in experimental tasks (Lewis, Derlega, Shankar, Cochard, & Finkel, 1997) and that videos of one interlocutor's gestures can affect a participant during experimental tasks, depending on the semantic similarity of linguistic referents (Mol, Krahmer, Maes, & Swerts, 2012), with other worries about confederates extant (Kuhlen & Brennan, 2013). Our study's preparatory phase included a norming experiment for conversational prompts and a thorough, tutorial‐style training program for the confederate to maximize the naturalism of the discussions for participants.

The video recordings were first qualitatively analyzed with manual annotations of gesture phrases for whether they conformed to any one of the six roles identified above, or if they could not be coded as one of them at all. Our independent variables (visual occlusion and conversation topic complexity) were designed to modulate the frequency of use of gestures to test the McNeil and Kendon philosophies of gesture (those first planned for analysis: iconic, metaphoric, pantomime, deictic, emblem, beat). For visual occlusion, we were uncertain about which gesture roles, and in which direction, frequency of use would change, given the understudied nature of gesture use during in‐person experiments involving a partition blocking torso visibility. That said, one might speculate that Kendon's position predicts a decrease in gestures during visual occlusion of the confederate's torso—if gestures are pragmatic *qua* intentional, speakers should gesture less when they know their gestures cannot be seen. A perfect Kendonite theory would suggest that gesture production should cease entirely, whereas a weaker pragmatic account would advocate for some kind of decrease. Contrariwise, McNeill's position might predict no change in gesturing: if gestures serve no (conscious) pragmatic purpose, participants’ gestures may not change between visual occlusion conditions as speakers continue to orchestrate their speech in the same way. For topic complexity, there is a similar lack of evidence suggesting whether people gesture more during more complicated conversations during in‐person interaction. Given that one study finds people gesture more when they have more ambiguities to resolve (Rasenberg, Pouw, Özyürek, & Dingemanse, 2022), we predicted that people may gesture more during complex topics, though we were not certain which gestures might increase.

These analyses were preregistered on the Open Science Framework (https://osf.io/7xbcd/?view_only=19f2b169f28f4d5c895c364cf7c0eebf). Following this first analysis in line with our preregistered protocol, which critically only considered six different roles of gesture, we undertook a second analysis to investigate whether those gestures that did not fall within any of these original six roles had interactive properties and applied our previous hypotheses and tests to those gestures. We make this clarification in the spirit of Open Science Framework practices. It is this second analysis concerning interactive gestures that constitutes the interest of the present paper because of their specific relevance to both conscious and unconscious philosophies of gesture, thanks to their analyzability solely as audience‐directed. This decision is nevertheless explicated through the preregistered plan performed. Extending inferential tests to the other gesture roles would not adjudicate between the theoretical accounts under investigation. Accordingly, analyses of interactive gestures should be understood as a theoretically motivated extension beyond the preregistered confirmatory analyses, rather than a replacement of them. Resultantly, this paper is presented as having two goals: its original, to test fundamental philosophies of why we gesture, and its follow‐on, to highlight the interactive role gestures play, in light of our preregistration's failure to include them, the literature's generally limited treatment of them and given their analyzability only as audience‐directed—making them uniquely placed to probe our original goal.

## 2 Methods

### 2.1 Participants

Fifty‐two monolingual British English speakers were recruited from the staff and student population of our university and were paid a £10 voucher for their participation. Four participants were excluded from analysis; three for revealing post‐test nonconformity with exclusion criteria (British English monolingual adult with no history of impairment in the capacities to speak, move, or hear) after the experiment was completed (one bilingual, two neurodivergent) and one for data quality issues concerning the placement of the visual occlusion revealed at the analysis stage. This left 48 participants who were included within qualitative analysis (M_age_ = 39, Range_age_ = 19–64; M_YoE_ = 17, Range_YoE_ = 11–22; Sex: 20M, 28F). Importantly, we recruited participants from a wide range of ages, as recent calls for better quality psychological science encourage (Majid, 2023). Participants gave written informed consent, and the experimental design was approved by the university's ethical approval committee. Revelation of the deception involved in the experiment occurred once the experimental procedure was completed. No participant reported they had the realization that their interlocutor was a confederate, suggesting a robustness to the dyadic conversations as being natural (as far as natural is possible in an experimental room).

### 2.2 Materials

Cards with questions on them were presented to participants in the second stage of the procedure (see Section 2.3). These were included as an attempt to add experimental control over the otherwise unconstrained nature of a conversation. Conversational paradigms have been used in gesture research before (Nota, Trujillo, & Holler, 2021; ter Bekke, Drijvers, & Holler, 2020; Trujillo et al., 2021), but their designs have always had participants converse unguided or unprompted, or else given a loose theme. While this is reasonable in principle, the suggestion that participants can “simply talk as they would if they met at home or in a café for the duration of the experiment” (Trujillo et al., 2021, p. 647), may arguably be contradictory given that the environment of the experiment in which participants talk is physically, socially, and emotionally different than that of one's home or a café in which one might have a chance encounter. Moreover, being instructed to discuss (dis)agreements about “privacy,” “social media,” or “language in teaching” (Nota et al., 2021, p. 4; ter Bekke et al., 2020) has limited generalizability to everyday dialogue. We suggest that naturalistic conversation may be more closely approximated in the laboratory with unstrict prompts—questions which participants are told can guide dialogue but need not be strictly adhered to or answered. Giving a range of topics as questions, about which they are permitted to speak on, around, or past, prevents situations of social awkwardness where interlocutors have nothing to say to one another, while not constraining participants to complete some kind of objective, as is required in task‐like conversational prompts used previously, but rather give themselves to the conversation.

These questions underwent rigorous norming to ensure experimental suitability—so as to prevent any social awkwardness while ensuring each question was easy to read, understand, and feel excited or interested about answering. Materials trialed in the norming study consisted of question sentences under six topics (hobbies, travel, food, politics, ethics, and life and death) within two complexity categories (simple, the former three topics, and complex, the latter three topics) generated by the lead author. One hundred and twenty English‐speaking participants were recruited from Prolific (Prolific, 2014, www.prolific.com) and assessed in Qualtrics (*Qualtrics*, Provo, UT) in a 6×4 between‐subjects factorial design with two independent variables (conversation topics and feature) across six levels for conversation topic (travel, hobbies, food, politics, ethics, and life and death) and four levels for feature (readability, answerability, predicted expressiveness, and complexity). Readability and answerability were chosen to ensure participants in the experiment were not conversationally inhibited by the questions themselves. Predicted expressiveness was invented to avoid participants being bored and, if possible, facilitate gestural productions; gesture expressiveness has been correlated with more emotional resonance (Rühlemann & Trujillo, 2024). Complexity was included to create a contrast of simple and complex question types for counterbalancing the perceived social or emotional intensity of conversational topics (see below). Manipulation of the complexity variable motivated the usage of a confederate in the assumption that controlling for and thus minimizing social friction between interlocutors would facilitate participants’ feeling sufficiently comfortable to share responses on deeper matters, such as life and death. No participant refused to answer any such difficult questions, nor cited discomfort with their interlocutor such that they did not wish to speak on a specific matter.

Each feature was defined in simple terms and two examples of opposite value (high and low in that feature; order of value counterbalanced across features) in that feature were presented as attention checks. No example question was included within the main stimulus set, and participants who rated a low‐value example question highly (or vice versa) were requested they return their submission (equivalent to excluding in Prolific). Norming study trials were headed by a feature question that expounded the meaning of the feature without using the term the feature was introduced with, to avoid participants generalizing from jargon as a result of misunderstanding. The dependent variable was a Likert scale rating from 1 to 7 for each question within the specific topic for a given[Table cogs70195-tbl-0001]feature.

**Table 1 cogs70195-tbl-0001:** Features rated for questions presented in the norming study, plus how each feature was defined to participants, how each was probed with a prompting question per trial, and the two example questions presented per feature to test understanding and act as an attention check

Feature	Definition	Feature question	Examples
**Readability**	“**Readable**” refers to **how easy it is to read** the question, but **not how easy it is to understand**. This concerns the **ease of parsing** (**putting together the words** in an order that makes a sentence), but not how easy it is to understand that sentence. For example, if you find yourself **having to read the question** again because the order of the words did not make sense to you, **then it is not very readable**.	How easy is this question to read?	What is your favorite snack?
			Did the old man the fortress?
**Answerability**	“**Answerability**” refers to **how long it would take** to answer the question. This concerns **how immediately an answer would come to you**, regardless of whether that answer is **basic or more profound**. For example, if you would be able to answer the question **without spending too much time**, even if you have lots to say or something deep to say, it is **highly answerable**.	How quickly would you be able to answer this question?	What is the square root of four hundred and three?
			How often do you brush your teeth?
**Predicted expressiveness**	“**Predictably expressive**” refers to how **expressively you might respond to this question** if you were asked it **in real life**. This concerns how much **passion** you might display, how **animated** you might get, or whether you imagine yourself **gesturing to convey your point**. If you struggle to picture how you respond, think about how others (who you know well) might describe how expressively you would respond. For example, if you could see yourself **answering with lots of gestures**, or you can see **someone you know well saying that you would answer passionately**, then it is **highly predictably expressive**.	If you were asked this question in real life, how expressively would you respond to it (from your perspective or someone who knows you well)?	Is grass green?
			Are politicians and businesses doing enough to prevent climate change?
**Complexity**	“**Complex**” refers to how complex **the topic under discussion** is, but **not how complex the language in the question is**. This concerns whether the **issue, problem, or general topic** being proposed is quite simple and basic or whether it is **more complex and profound**. For example, something would be **simple** if it requires just **a bit of world knowledge, a single statement of fact, little debate, or not much thinking** to answer.	How complex does this topic seem to you?	How does the brain repair itself after a stroke?
			How many legs does a dog have?

*Note*. Items in bold are as presented to participants.

For this norming, G*Power (Faul, Erdfelder, Lang, & Buchner, 2007) recommended a sample size of 45 participants to rate questions on all features for a power of 0.95124 at α = 0.05 in a t‐test comparing the difference between two dependent means from matched pairs. Given that, for all 60 questions, there are four features that require 45 individual responses, this produced a total of 10,800 responses to be collected. To achieve this with the procedure detailed above, 180 total participants would be required. Due to budget constraints, the maximum number of participants achievable was 120, meaning that 30 participants rated all questions for a given feature, leading to a power of 0.8482542 at α = 0.05 in a t‐test comparing the difference between two dependent means from matched pairs.

Questions were selected from the norming study when their predicted expressiveness and answerability scores were above a mean of 4.5. This led to 15 topics being eligible. To allow for at least two questions per topic, the same number of questions per complexity level, one additional question was selected from the life and death, food, and travel topics—this also led to a balance of two topics per complexity level with four questions, two topics with three questions, and two topics with two questions. This final step allowed a total of 18 eligible questions, which are presented below. According to an independent samples *t*‐test, complex questions were rated significantly more complex than simple questions (*t*(58) = –12.00, *p* < .001)[Table cogs70195-tbl-0002].

**Table 2 cogs70195-tbl-0002:** Questions selected for inclusion in the experiment, with which topic and complexity level they pertained to, with their readability (“Read.”), answerability (“Ans.”), predicted expressiveness (“Pred. Ex.”), and complexity (“Comp.”) mean scores from 1 to 7

Topic complexity	Question	Topic ID	Read.	Ans.	Pred. Ex.	Comp.
**Simple**	Where are the nicest places you have been around the world?	Travel 1	6.41	5.61	4.88	2.46
	What locations would you like to visit next?	Travel 2	6.48	5.39	4.88	2.54
	Are babies too young to fly on airplanes?	Travel 3	6.00	5.11	4.24	2.75
	What is your favorite meal?	Food 1	6.84	5.52	5.03	2.04
	Does pineapple belong to pizza?	Food 2	6.94	6.52	4.10	1.96
	What is fun to do when you are bored?	Hobbies 1	6.04	5.17	4.58	2.30
	What activities do you do during free time?	Hobbies 2	6.39	5.83	4.81	2.04
	Do you have a favorite book?	Hobbies 3	6.96	5.13	4.55	2.15
	Is there something you have always wanted to try but never got the chance to?	Hobbies 3	6.39	5.50	4.81	2.89
**Complex**	Could anything push you to consider an assisted death?	Life & Death 1	6.03	4.50	4.83	4.89
	Is there anything more important than love?	Life & Death 2	6.83	4.43	4.52	4.36
	Would you support military action against a country that invaded a close UK ally?	Politics 1	5.69	4.52	4.84	5.45
	Would trains be better managed by government than by private companies?	Politics 2	5.88	5.04	4.50	4.93
	Should big businesses have to pay more tax?	Politics 3	6.85	4.92	5.34	4.45
	Should all countries have to reduce emissions equally to prevent climate change?	Politics 4	5.69	4.92	5.03	4.93
	Do people deserve a second chance?	Ethics 1	6.68	5.22	5.33	4.07
	What rights do humans deserve?	Ethics 2	6.24	4.56	5.63	4.40
	Should you always obey authority?	Ethics 3	6.24	4.52	5.03	4.90

These questions were printed on hand‐sized cards and presented in a pseudorandomized order to participants, such that the pack of cards with the questions were ordered simple, then complex, then simple, and so on, but that no order of specific questions was prescribed. No experimental session answered all 18 questions.

### 2.3 Procedure

Prior to the experiment beginning, we recruited a confederate (a second‐year female undergraduate psychology student) who underwent a detailed onboarding and tutorial‐style training procedure to familiarize them with the experiment and gesture studies. Onboarding involved testing their understanding of the study procedure and completing piloting of it with the lead author. Given the use of topic cards and the screen, it was not possible to blind the confederate completely from experimental hypotheses. Regularly during the 3‐month data collection period, tutorial‐style training involved detailed paper discussion of key works in the theory, psychology, and neuroscience of gesture (examples include: Chui, Yeh, & Chang, 2021; Holler, Kendrick, & Levinson, 2018; Joue et al., 2020; Kendon, 2004a; McNeill, 1992; Rasenberg et al., 2022). This thorough training strengthened the experimental team's confidence in the confederate to lead dyadic conversations in a socially frictionless manner without overly biasing the participant's responses. Our use of the confederate kept to all recommendations (Kuhlen & Brennan, 2013) as far as was possible or applicable.

Participants were first introduced to the study environment by the lead author, where the nature of their participation was explained (they were told that their thoughts on certain topics were required, critically, with no mention of gesture analysis), and informed consent was gained. This occurred with the confederate, simultaneously to ensure success in the deception. Once seated, a ceiling‐ or wall‐mounted camera (a Logi Webcam C920e) recording began capturing video and audio data of the participant, and the lead author sat in the corner of the room, not appearing to concentrate on the conversation by typing on a keyboard and reading a computer screen. Due to infrastructural constraints, the confederate's gestures were unable to be recorded. However, as mentioned, the confederate was highly trained for this study and was thus aware to ensure their gestures did not influence their interlocutor unduly, minimizing risk of compromising the naturalism of the experiment while simultaneously making them ineligible for their gestures to be recorded anyway.

**Fig. 1:**
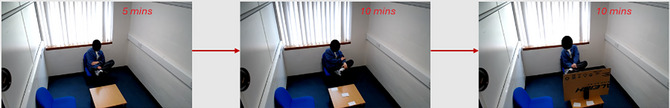
Visual representation of the experiment procedure in the warm up (left image—5 min, no cards, no screen), the no screen phase (central image—10 min, cards, no screen), and the screen phase (10 min, cards, screen). Note that the screen's face was blank on the participant's side.

The experimental period consisted of three strictly timed periods: a 5‐min “warm‐up” chat, a 10‐min conversation prompted by the cards at a self‐paced rate of question selection, and a final 10‐min duplicate of the second period, where a cardboard screen was placed between the interlocutors. These time periods were strictly adhered to, and conversations continuing at the end of a given period were interrupted. The screen occluded all of the confederate's body to the participant, apart from their head and vice versa, meaning also the torso and thus the area in gesture space (McNeill, 1992) in front of the body where gestures typically occur. This procedure is illustrated in Fig. 1.

The confederate began by prompting conversation naturally, asking about the participant's job, background, and other small talk matters not already covered by the topic questions. After exactly 5 min, the lead author interjected with the topic questions and placed them in front of the confederate and participant on a table, explaining that they should discuss the topics on them. The confederate was always selected first to implicitly model the card selection process—picking the card up, reading the question aloud, placing it on the table in between the participants, and only then beginning to discuss—to ensure cards were not continuously held by participants and thus impeding gesture analyzability. The confederate also ensured that any conversation that seemed to come to an agreement or end was replaced via a new question. As mentioned, no participant reported awareness that their interlocutor was a confederate during the debriefing—anecdotally, many were deeply surprised, speaking well to the conversations’ naturalness.

### 2.4 Analyses

Qualitative analyses of manual gestural productions produced by participants (but not the confederate) were analyzed using ELAN (ELAN, 2024). Gesture roles were coded as one of seven possible annotations: iconic, metaphoric, pantomime, beat, deictic, emblem, or otherwise uncoded. A gesture phrase was considered to start at the preparation for the stroke and conclude at the end of the retraction (Kendon, 1980). For sequences of gesture phrases, where the hands did not rest between gesture roles being realized and thus where no obvious retraction occurred, gesture phrases were considered those that started with preparation and contained an identifiable stroke. Our coding schemes for specific gestures from the first (the first six) and second (interactives) analyses are defined in Table 3. Gestures that did not fall into any of the first six roles were initially classified as “uncoded.” Self‐adaptors—for example, adjustments of the hair—were not coded as gestures.

**Table 3:** Coding scheme for all seven gesture roles annotated in this experiment

*Gesture role*	*Coding scheme definition*	*Example*
*Iconic*	Where the form or motion of the hands attempts to visually represent a property of a literal referent explicit or implicit from speech.	An oscillating downward motion starting high to convey the wavy nature of a road descending a mountain.
*Metaphoric*	Where the form or motion of the hands capture the source domain of a metaphor whose target is realized during speech.	The hands with palms facing one another held outward at the width of the body to symbolize the “size” of an idea.
*Pantomime*	Where the form and motion of the hands mimic the use of an object or tool. Distinguish from iconics where the literal referent in speech does not have a visual property embodied by the hands.	Hands held outward with palms downward and the fingers moving upward and downward to symbolize typing on a keyboard
*Beat*	Where motions of the hands are temporally synchronized with lexical, semantic, or suprasegmental landmarks in speech.	A clasped fist rising and falling on every word or syllable
*Deictic*	Where forms and motions of hands are directed at particular points in space around the speaker to make reference to specific points in time, abstract or concrete points in space, ideas under discussion, and the interlocutor.	A pointed forefinger marking a position to the left of the interlocutor to establish the reference point for a person or place under discussion.
*Emblem*	Where forms and/or motions have identifiably conventional or lexical meanings, be those meanings explicitly conventional or implicitly lexical.	A thumbs up for “good” or a flat hand shape with fingers outstretched and a left‐right‐left motion to indicate negation.
*Interactive*	Where the hand makes some motion or adapts form in some way to direct toward the confederate without pointing at their person, primarily for intersubjective acknowledgment or information transfer.	An outstretched palm‐up with co‐occurring speech appearing to give reassurance to the confederate.

A second analysis was prompted of the uncoded gestures upon the lead author completing the first analysis. It became transparent that many of these took forms involving an outward motion of the participant's hand in the direction of the confederate. This unexpected finding prompted a second pass of the data beyond preregistered plans, where qualitative analysis was completed solely on the uncoded gestures to code them as interactive or not. Understanding of interactive gesture types was informed by Bavelas et al. (1992) and adapted based on the lead author's observations throughout the first analysis. In this, interactive gestures were broadly those where the participant seemed either to acknowledge the existence of some internal mental contents of the confederate or where there was some motion with the hand to transfer to the confederate the information communicated in speech simultaneously.

Qualitative analysis of gestures was built upon existing best practice (Kong, Law, Kwan, Lai, & Lam, 2015) for assessing codes’ reliability by involving a quality control stage over around half of gesture tokens before reanalysis for inter‐rater reliability on nearly a fifth of all gestures. First, the lead author completed the first analysis. Then, data from 17 out of 48 participants, 4091 out of 8309 gestures (49.2%), underwent quality control by a research assistant trained in gesture annotation. Such a step did not solely aim to determine the accuracy of code annotation but also to ensure gesture phase boundaries were correctly placed and that no specific gestures were missed (e.g., within a longer gesture phrase where a quick, under 1‐s long beat may have been incorporated into a different gesture). Second, author AEP was trained on nonparticipant gesture annotation examples and data from two participants before independently completing ratings for a different set of 13 out of 48 participants (randomly selected), 1574 out of 8309 gestures (19%). Inter‐rater reliability for gesture role coding was high, with a Cohen's Kappa of 0.854, a percentage agreement of 88.8% (1398 of 1574 items), and a statistically significant result (*p* < .001). Analysis of uncoded gestures as interactive or not was completed by the lead author, which was also checked for percentage agreement by a research assistant who analyzed a subset of gestures coded as interactive (1071 of 2103) for their status as interactive gestures, revealing substantial agreement (82.1%).

Data for the frequency of gesture roles are reported as descriptive statistics with percentages. Statistical analyses for the change in interactive gesture incidence depending on the presence of the screen were conducted with a paired sample *t*‐test. Testing the influence of topic complexity on interactive gesture frequency leveraged a linear mixed‐effects model. Inter‐rater reliability was calculated with Cohen's Kappa. These were completed using jamovi (The jamovi project, 2025) and R (R Core Team, 2025).

## 3 Results

Across all three phases of the experiment, the 48 participants produced 8309 manual gestures. Proportions of all gesture roles are reported in Table 4. These data revealed that the most common role of gesture is deictic, with uncoded as the second most common code at 14 fewer gestures. Beats and metaphorics were next most common, followed by iconics, emblems, and pantomimes.

**Table 4:** Frequency and percentage of each gesture role coded for in the first analysis

*Gesture role*	*Frequency*	*Percentage*
Deictic	2370	28.52%
Uncoded	2356	28.35%
Beat	1359	16.36%
Metaphoric	1345	16.19%
Iconic	515	6.20%
Emblem	183	2.20%
Pantomime	181	2.18%
***Total gestures***	***8309***	

**Fig. 2:**
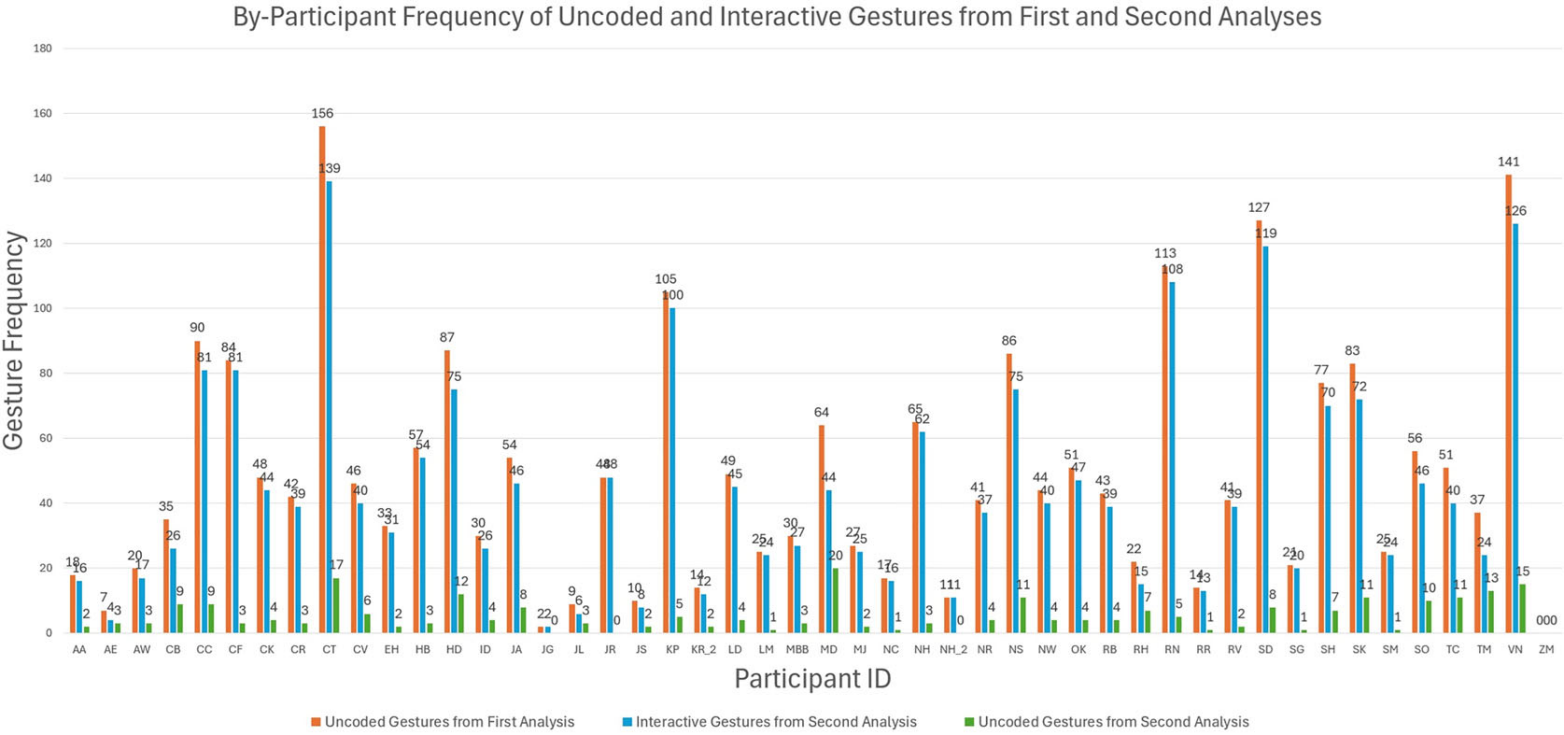
By‐participant data for total gestures, total uncoded gestures, and how many of those uncoded gestures were coded as interactive in the second analysis.

Results of the second analysis, coding every uncoded gesture as interactive or otherwise uncodeable, are presented overall and by participant in Fig. 2. By‐participant data are included specifically to illustrate the variability of total interactive gestures produced and the consistency of the percentage of uncoded gestures that were uncodeable as interactive across all participants. This analysis revealed that 89.26% of all uncoded gestures were classified as interactive, a total of 2103, leaving 10.74% or 253 uncoded gestures as uncodeable across all seven possibilities. This meant that, of all gestures, 25.31% were interactive and 3.04% were uncoded.

Upon conducting a paired sample *t*‐test to assess by‐participant changes in interactive gesture incidence during the screen and no screen phases, the Shapiro−Wilk test indicated that the assumption of normality for the difference scores was violated (*W* = 0.940, *p* = .017), so a nonparametric Wilcoxon signed‐rank test was conducted. This revealed a statistically significant reduction in the number of interactive gestures when the screen was present compared to when it was absent (*W* = 752, *p* < .001). For comparison, the paired‐samples *t*‐test also showed a significant effect, *t*(47) = 3.68, *p* < .001. See Table 5 for by‐question and total interactive gesture frequencies per topic complexity level.

**Table 5:** By‐topic‐question frequences and totals of interactive gestures made during the no screen and screen phases of the experiment

	*Simple topic questions*									
*Phase*	*T1*	*T2*	*T3*	*F1*	*F2*	*H1*	*H2*	*H3*	*H4*	*Total*
No screen	40	67	82	93	49	80	71	40	36	*558*
Screen	19	24	37	18	31	60	64	37	33	*323*
**Total**	**59**	**91**	**119**	**111**	**80**	**140**	**135**	**77**	**69**	***881***

	*Complex topic questions*									
*Phase*	*LD1*	*LD2*	*P1*	*P2*	*P3*	*P4*	*E1*	*E2*	*E3*	*Total*
No screen	56	11	20	58	42	80	64	43	42	*416*
Screen	75	43	56	24	38	22	38	34	57	*387*
**Total**	**131**	**54**	**76**	**91**	**80**	**102**	**102**	**77**	**99**	***803***

*Note*. Consult Table 2 for the full length names of topic question IDs.

**Fig. 3:**
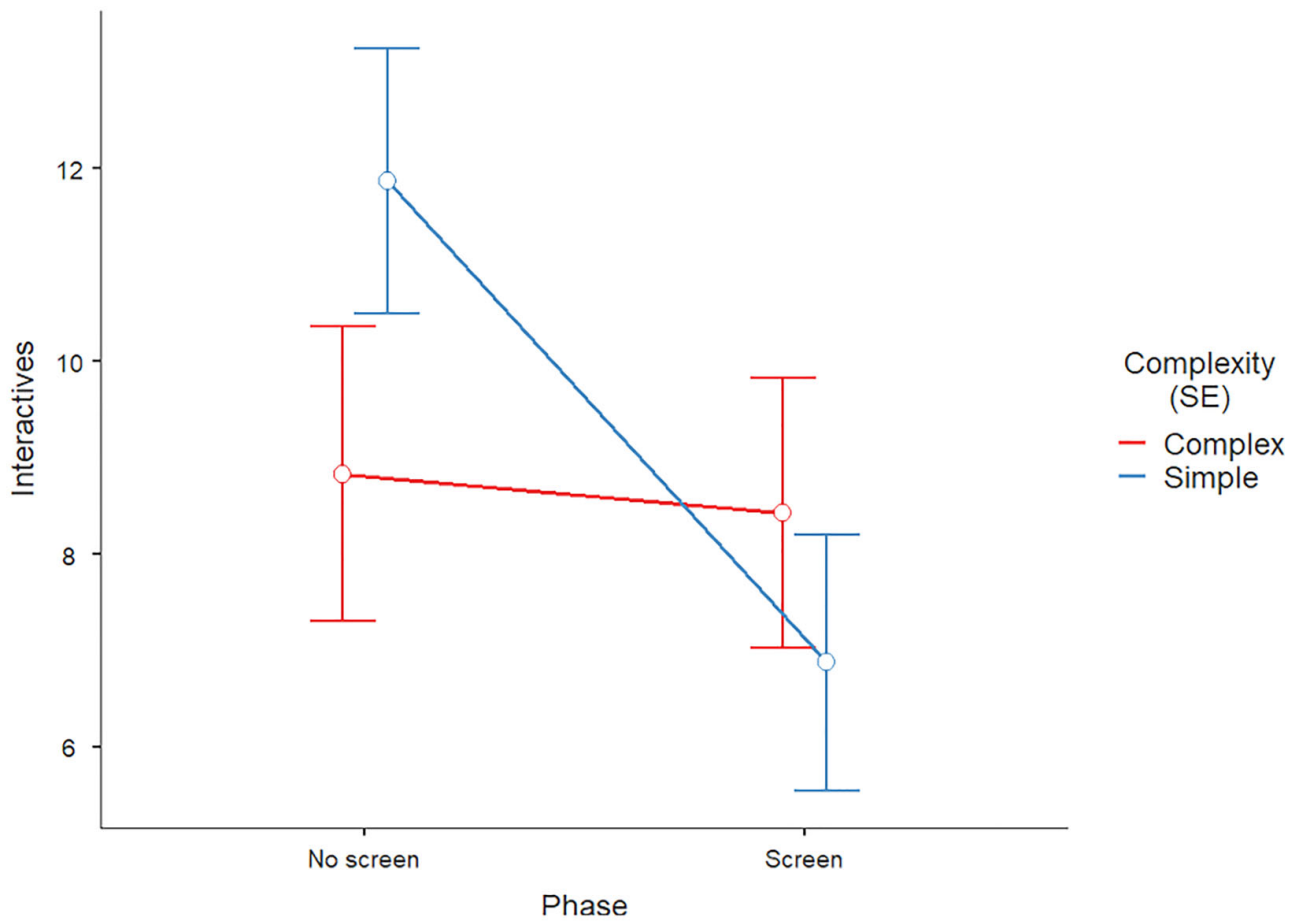
Change in frequency of interactive gestures produced during no screen and screen phases of the experiment by their production within simple or complex conversation topics. Error bars indicate the model's standard error.

We also tested the interaction between participants’ interactive gestures, which phase of the experiment they made them within, and whether the conversation topic was simple or complex at that time. A linear mixed‐effects model examined how topic complexity and the presence of a screen influenced participants’ production of interactive gestures. The model included fixed effects of Phase (no‐screen, screen), Complexity (simple, complex), and their interaction, with by‐participant random slopes for Phase and Complexity [(0 + Phase + Complexity | Participant)]. Residuals were normally distributed, and the model converged without warnings (optimizer = bobyqa). Model comparison with a random‐intercept‐only model improved fit, χ^2^(6) = 15.36, *p* = .018, conditional *R* ^2^ = .22, marginal *R* ^2^ = .07. There was a significant main effect of Phase, *F*(1,86.4) = 8.73, *p* = .004 (as found above), *b* = −2.70, 95% CI [−4.51, −0.89], but no main effect of Complexity, *F*(1,46.6) = 0.39, *p* = .535, *b* = 0.75, 95% CI [−1.60, 3.09]. Importantly, the Phase × Complexity interaction was significant, *F*(1,92.9) = 6.57, *p* = .012, *b* = −4.59, 95% CI [−8.12, −1.06]. As shown in Fig. 3 (plotting estimated marginal means), the presence of a screen reduced interactive gestures primarily for simple topics (by ∼4−5 interactive gestures per participant), whereas gesture frequency for complex topics remained relatively stable across phases.

Explanation of this result is enabled with by‐question descriptive statistics of how many interactive gestures were produced by participants during the no screen and screen phases of the experiment. This is detailed in Table 5, with the critical comparisons being those where the amount of interactives is greater in the screen condition than in the no screen condition for complex topics. These are both Life & Death questions, as well as one Politics and one Ethics question. As totals show, there are overall more interactive gestures produced during complex topics in the screen phase than during simple topics in the screen phase. Fig. 3’s basis in the model's estimated marginal means, not raw totals, explains why visual inspection of interactive gestures during complex topics appears to be slightly lower in the screen phase.

## 4 Discussion

### 4.1 Main findings

We find that deictics make up the most common role of gesture used by our participants, but that an almost identical percentage of total gestures, as deictics, do not fall under any of these six. We also find that beats and metaphorics are almost as equally common as one another, that iconics make up around one twentieth of all gestures, and that emblems and pantomimes are as similarly infrequent.

The revelation of the proportion of gestures and their apparent interactive intention that were uncodeable led to the second analysis, determining how many of these uncoded gestures were interactive. We find that 89.26% (2103 of 2356) of uncoded gestures were interactive, and thus that 25.31% (2103 of 8309) of all gestures were interactive.

Our experimental design involved a change to the naturalistic setup of the dialogue: placing a screen between participants. We find that participants used interactive gestures significantly less frequently when the screen was put up compared with before, though this effect was driven by a reduction in visually occluded interactive gestures made during *simple* conversation topics. Importantly, complex topics yield a similar frequency of interactive gestures when the hands are occluded as when they are visible to the interlocutor.

### 4.2 The kinds of interactive gesture

During our first analysis, we observed that many gestures produced during interaction could not apply to our original six “roles” of gesture. These took no representational quality, did not seem to point to any discourse referent or physical object (lest their interlocutor), had no conventional meaning, and were not temporally or semantically synchronized with speech. Our coding scheme allowed cases such as these to be classified as “uncoded,” so as to preserve the integrity of analyses of the gesture roles for which judgments were more certain. Indeed, we found that uncoded gestures took a similar kinematic form to the description in Bavelas et al. (1992). This refers to the speaker extending the hand with a palm somewhat facing upward with fingers directed toward their interlocutor. They report that interactive gestures have four main functions: citing the other's previous contribution; seeking agreement, understanding, or help; the delivery of new versus shared information; and events around the speaking turn. We used this to establish two broad categories of interactive gesture in order to simplify coding: acknowledgment (e.g., of your interlocutor's point) and transferring (e.g., of information you are communicating verbally). Because these two categories are often kinematically indistinguishable—their ascription to a gestural form would be contingent upon knowing a speaker's intention—we simply code uncoded gestures as “interactive” or “N/A.”

However, it is our observation that linguistic context can provide clues to speakers’ intended uses of interactive gestures. Some interactive gestures were pro‐speech (i.e., not co‐occurring with the speaker's speech), which would make ascription as “transferring” difficult to justify. In these contexts, it might be sensible to suggest the gesturer is acknowledging their interlocutor's point of view as truthful or valuable. Similarly, some interactive gestures co‐occur with spoken content already in the conversation's common ground (Holler, 2010), thereby “acknowledging” or referring to their interlocutor's knowledge of the content so as not to appear to presume their interlocutor's unawareness.

In an effort to collect our 2103 observations of interactive gestures into a framework for future categorical analysis, we suggest that “acknowledgment” and “transferring” may take seven different kinds. We wish to stress that these kinds exist as generalizations over our observation of thousands of gestures that must undergo future testing to ensure construct validity. To be more specific, acknowledgment may be successfully split into four kinds: (1) of previous content of any participant; (2) information that is in the common ground; (3) a response from the confederate that is anticipated; or (4) a communicative intent of the confederate. Transferring may have three further distinct kinds: (1) of information that is new because it is new; (2) an adverbially styled operation over an opinion, such as softening or conceding; or (3) a request for approval or agreement with a perspective. It is not yet clear to us whether these kinds are uniquely ascribable to interactive gestures in the same way that this paper's first analyses involved the unique ascription of the above six legacy gesture roles. Further work, which may require experimental involvement of participants in judging their intentions behind their own gestures, is required to better understand interactives’ kinds.

### 4.3 A subconscious account for why we gesture

As mentioned above, differing opinions exist in basic gesture research as to why humans gesture. On one side, Kendon (2004b, 2017) argues that gestures serve a pragmatic role; that gestures represent an intentional effort to contribute to communicative goals. This is borne out via evidence from studies showing that communicative gestures are perceived by others to be meant as communicative in comparison to other kinds of bodily movement performed while speaking (e.g., fixing one's hair). On the other side, McNeill (1992, 2015) argues a more philosophical position. For McNeill, gestures are fundamentally an unconscious expressive embodiment of conceptual content; that we imbue our gestures with our Heideggerian (1962 [1927]) “Being” which serve to inhabit (Merleau‐Ponty, 1962) and ultimately orchestrate our speech. Evidenced by analyses of people retrospectively narrating cartoon videos, on this account, gestures intertwine with language to co‐express meaning in a way that allows people to represent their inner consciousness and relate to their external environments simultaneously.

To tease apart this apparent contraposition, our experiment included a semi‐naturalistic phase that inhibited the possibility of participants’ gestures being seen. After 10 min of question‐guided conversation, a screen was placed between the participant and the confederate for a further 10 min.

Were our conclusions drawn from data about all gesture roles analyzed, one might argue that visual occlusion of gestures minimally modulates audience‐directedness of gesturing, not conscious awareness of gesturing. This is why our analyses in this paper prioritized interactive gestures, departing from preregistered analyses. Interactive gestures’ purpose is to interact, thus they are always analyzable as being audience‐directed.

We thus subjected by‐participant data only for the frequency of interactive gestures in the no screen and screen phases. We find that participants gesture significantly less during the screen phase compared with the no screen phase, but that participants do still gesture interactively when the screen is present. Moreover, we find that this decrease in visually occluded interactive gestures pertains mainly to those performed during the discussion of simple conversation topics; complex topic interactive gestures stay stable during the no screen and screen phases. Taking solely the significant decrease from before to after visual occlusion, this might be taken as a result supporting a weak Kendonite position that (at least interactive) gesturing is intentional. However, the finding exists within the context of the experimental setup of participants being unaware of our analysis of their gestures,^2^ from which we might infer that their gesturing was not consciously modulated (at least across experiment phases). And yet, when the screen was up, participants invisibly directed interactive gestures at the confederate. Because there is no rationale for analyzing audience‐directed gestures as self‐directed, we must conclude that participants were not consciously aware of their occluded interactive gesturing (even though they may have become aware with post‐hoc introspection).

A core problem about understanding why humans gesture is in reconciling these two positions. It is true that we often are not aware of our gesturing, and often use it to orchestrate our speech (we find beats comprise 16.36% of gesturing) and as a kinematically embodiment of linguistic meaning (we find representational gestures comprise 24.57% of gesturing). We also clearly attempt to interact with our hands when our interlocutors cannot see them. However, these are not the only, or even predominant, roles of gesture (pointing and interacting comprise 53.83% of gesturing). Further, it is entirely possible to become conscious of what and why we gestured something through introspection; evidencing processes available to conscious intention that truly unconscious processes (e.g., homeostatic) cannot permit. The problem statement is thus: to gesture cannot be unconscious if introspective efforts permit access to retrospective awareness of gestures’ intentions, but it cannot be conscious if we attempt to interact with our hands when they are occluded to our interlocutors without awareness.

As hypothesized in the Introduction, the McNeill position might argue that gesturing cannot be conscious, also because our finding relating to topic complexity: the lack of a reduction in interactive gesturing with occluded hands during the screen phase only while discussing complex topics. Were this finding in relation to all gestures, it might be argued that self‐directedness serves as explanatorily useful; in that case, one can simply argue that gestures are being used by the participant to aid the cognitive task of communicating about a more complex topic (Goldin‐Meadow, Nusbaum, Kelly, & Wagner, 2001; Hostetter & Bahl, 2023; Morsella & Krauss, 2004). However, crucial to our analysis is the focus on interactives specifically, being those gestures that are directed to acknowledge intentions of or transfer information to the interlocutor (Bavelas et al., 1995; Williamson et al., 2025). In this case, we argue that the persistence of the urge to engage with the interlocutor with the hands *only during complex topics* may reflect an override of visual‐feedback inhibition (given the hands’ invisibility) by social‐politeness demands for intersubjective acknowledgment (Brown & Levinson, 1987; Tantucci, 2021).

Complex topics in this experiment often included those that were more emotionally challenging to answer (irrespective of affect valence), and we interpret these findings thus as being driven by those questions that particularly demanded participants go above and beyond with politeness. This is borne out in by‐question observation of Table 5, with both Life & Death questions “Would anything push you to consider an assisted death?” (*n_interactives|no screen_* = 56, *n_interactives|screen_* = 75) and “Is there anything more important than love?” (*n_interactives|no screen_* = 11, *n_interactives|screen_* = 44) yielding more interactives while the screen was present, as well as “Would you support military action against a country that invaded a close UK ally?” (*n_interactives|no screen_* = 20, *n_interactives|screen_* = 56) and “Should you always obey authority?” (*n_interactives|no screen_* = 42, *n_interactives|screen_* = 57) from the Politics and Ethics topics, respectively. No other complex topics followed this pattern, nor did any from the simple group. Thus, the problem of arguing that interactive gestures are “conscious” when we unwittingly interact with our hands when they are occluded to our interlocutors most poignantly applies to these cases. With complex topics, there is apparently such an urge to be socially cohesive through expressing politeness with intersubjective acknowledgment that it overrides any possibly conscious thought not to bother with gesturing (given its occlusion).

We interpret these findings of visual occlusion and topic complexity collectively, which suggest a truth to both sides, really to evidence a third position advocated first in Wolff (2015 [1945]), but never considered in contemporary gesture research: that gesture is *sub*conscious. The case for the Kendonite position is made stronger by the significance of our comparison between interactive gesturing during the no screen phase versus the screen phase—people gesture interactively significantly less when their gestures are not visible. And yet, McNeill's position is still tenable given the nonzero nature of occluded interactive gestures used and their persistence during visually occluded complex conversations—even when one's hands are not visible, one still manually gestures to interact with one's interlocutor. A fortiori, what is betrayed by the case of topic complexity preserving interactive gesturing while occluded is a socially (*qua* politeness through intersubjective acknowledgment) motivated propensity to gesture that apparently bypasses conscious thought (*qua* the cognitive inhibition one might expect a gesturer to display so as not to use an occluded modality of communication).

In this way, our data permit recourse to the explanation so literarily put by Wolff (2015 [1945], p. 4): “gestures arise from the subconscious mind without passing through the cooling temperature of the critical consciousness.” While Wolff's usage of “subconscious” refers to the revelation of emotional states that gestures may facilitate, we explain gesture by suggesting that it comes from intrinsic communicative motivations within the subconscious. The term “subconscious” may appear pseudoscientific, but studies investigating priming and automaticity, robustly established psychological constructs, presuppose a behavioral guidance system unavailable to immediate conscious awareness in the same way (Bargh & Morsella, 2008). Our hypothesis is the speculative proposal that two key subconscious motivations drive gesturing: *to express oneself* (which our politeness propensity may evidence) and *to be understood* (into which further work may be required).

### 4.4 Implications and limitations

We explain our focus on interactive gestures through our preregistered protocol and our motivation behind the post‐hoc analysis into interactives specifically (the high number of gestures not codeable as iconic, metaphoric, pantomime, beat, deictic, or emblem). It is important to caveat that while our original coding scheme's absence of the “interactive” role explains the extent of uncoded gestures during our first analysis, there remain uncoded gestures (253 gestures, or approximately 3% of the database) following our second analysis. Given the uncodified nature of gestural units (in contrast with speech units, which are graphically codified in written languages, for example), one hopes that 3% of the dataset being uncodeable falls within a reasonable threshold for kinematic ambiguity. That said, future work could incorporate quantitative kinematic analyses to reduce such ambiguity where other blockers to clear analysis are not present (e.g., an untransparent link between handshape and language).

In our experiment, we recorded frequencies of use for the six main types of gesture investigated in gesture research. One particular type of gesture predominates scientific study: iconics. Iconic gesture has been the explanandum of a wealth of different research methodologies, including formal semantic, behavioral, neurophysiological, neuroimaging, comparative cognition, and developmental (Beattie & Shovelton, 2001; Chui, Lee, Yeh, & Chao, 2018, 2021; Cocks, Morgan, & Kita, 2011; Dick, Mok, Beharelle, Goldin‐Meadow, & Small, 2014; Green et al., 2009; Kandana Arachchige, Simoes Loureiro, Blekic, Rossignol, & Lefebvre, 2021; Krason, Fenton, Varley, & Vigliocco, 2022; Lücking et al., 2024; Masson‐Carro, Goudbeek, & Krahmer, 2017; McNeill, 1986; Ortega & Özyürek, 2020; Özçalişkan, Gentner, & Goldin‐Meadow, 2014; Schlenker, 2019; Tanner & Byrne, 1996; Wu & Coulson, 2005, 2007; Yap, So, Melvin Yap, Tan, & Teoh, 2011). We find that iconic gestures make up 6.2% of all gestures produced by our participants: healthy, neurotypical, monolingual British English adults with a mean age of ∼39. If one takes frequency of use as a yardstick for importance to scientific study, iconics must be placed lower than deictics, interactives, and metaphorics, for example. While this argument does not necessarily hold, we contend that there are overlooked properties of these three types of gesture that an overemphasis on iconics may miss. This paper attempts to address the absence of a closer investigation of interactive gestures, but future work must also take a closer look at the other gesture types we coded for and extrapolate their properties and kinds equally; especially given the unprecedented richness of our dataset.

Our study involved a participant conversing with a confederate in a laboratory environment, also for a portion, with a screen placed between them. In our discussion of results, we have presented findings as illustrative of gesturing in general, such as in actually naturalistic settings. A key presumption in our argument is that the notion of “nature” in conversational research is definable. As famously illustrated (albeit within moral philosophy), the term is impossible to pin down because in common parlance it refers both to everything that exists or that which is untouched by humans; but that which exists includes everything touched by humans, and that which is untouched by humans cannot be studied without being “touched” (Mill, 1874). A “naturalistic conversation” is thus impossible to record. In this way, we—like others studying naturalistic conversation—trade on an ambiguity for the benefit of scientific argument, and do not object in practice to the term's usage (though perhaps “everyday conversation” brings less metaphysical baggage). However, we believe our results are borne from good research design that means they are generalizable to inferences about how people gesture in their everyday lives. Our methodological choices aimed to better approach naturalness than previous studies (Nota et al., 2021; ter Bekke et al., 2020; Trujillo et al., 2021), although we concede this is practically impossible.

## 5 Conclusion

This study investigated the occurrence of gestures whose roles appear to be interactive—where speakers acknowledge their interlocutor's ideas or opinions, or signal the transfer of information to their interlocutor. We find that over a quarter of all gestures are interactive, despite comparatively little research into them versus iconic gestures, which account for around 1 in 20 gestures we analyzed. Participants used interactive gestures significantly less with their interlocutor when they could only see each other's heads—when torso, and thus gesture, visibility was occluded—compared with when no screen was placed between them. This effect only concerned interactives produced during simple conversations; complex topics preserved the frequency of interactive gestures into the visual occlusion condition. We suggest our findings advocate a middle ground between McNeill's and Kendon's philosophies of gesture, suggesting a revisiting to Wolff (2015 [1945]), that explaining why we gesture must involve recourse to subconscious motivators for being understood and expressing oneself.

## Funding

This research was supported by a doctoral scholarship awarded to the lead author by the University of the West of England.

## Conflict of interest

The authors declare no competing interests.

## Data Availability

Statistical data are available by request to the lead author. Videos of participants are not available for sharing.

## Notes

1 Note that metaphoric gestures are not iconics for *abstract* semantics, a common misconception about the nature of metaphor realization in gesture (Kita, Alibali, & Chu, 2017; Kong, Law, Kwan, Lai, & Lam, 2015; McNeill, 1992). Instead, they capture metaphorical semantics in which gestures constitute the source domain. This is important because iconic gestures can be abstract. This is explicated with a double dissociation. For iconics, you can represent abstract semantics with your hands as an iconic gesture; for example, a forefinger movement up and down when describing someone's commute on the Eurostar under the English Channel via the train tunnel to and from London. For metaphorics, a gesture's form and/or motion can comprise the source domain for a target related to spoken semantic content; for example, a horizontal hand wave spanning the width of the torso occurring simultaneously with speech concerning the totality of some phenomenon (e.g., “all services are offline,” underline signaling gesture co‐occurrence). Note that semantics are literal but what makes the gesture metaphorical is that the kinematic source (distance that the hand travels) embodies an unrelated semantic target (the array of services referenced).

2 To this point, over the ∼20 h of video footage, only once did one participant overtly acknowledge awareness of their own gesturing during speech.
